# Dissection of early transcriptional responses to water stress in *Arundo donax* L. by unigene-based RNA-seq

**DOI:** 10.1186/s13068-016-0471-8

**Published:** 2016-03-08

**Authors:** Yuan Fu, Michele Poli, Gaurav Sablok, Bo Wang, Yanchun Liang, Nicola La Porta, Violeta Velikova, Francesco Loreto, Mingai Li, Claudio Varotto

**Affiliations:** Department of Biodiversity and Molecular Ecology, Research and Innovation Centre, Fondazione Edmund Mach, Via E. Mach 1, 38010 San Michele all’Adige, Trento Italy; Dipartimento di Biotecnologie, Università degli Studi di Verona, Verona, Italy; Dipartimento di Scienze Agrarie, Università di Bologna, Bologna, Italy; Centro di Biologia Integrata (CIBIO), University of Trento, Trento, Italy; College of Computer Science and Technology, Jilin University, Changchun, China; MOUNTFOR Project Centre, European Forest Institute, Via E. Mach 1, 38010 San Michele all’Adige, Trento Italy; Institute of Plant Physiology and Genetics, Bulgarian Academy of Sciences, Sofia, Bulgaria; The National Research Council of Italy (CNR), Department of Biology, Agriculture and Food Sciences, Rome, Italy

**Keywords:** *Arundo donax*, Water stress, RNA-Seq, Abscisic acid (ABA) signaling, Transcription factors, Conserved drought-responsive genes, Poaceae, Osmoregulatory proline metabolism

## Abstract

**Background:**

*Arundo donax* L. (Poaceae) is considered one of the most promising energy crops in the Mediterranean region because of its high biomass yield and low input requirements, but to date no information on its transcriptional responses to water stress is available.

**Results:**

We obtained by Illumina-based RNA-seq the whole root and shoot transcriptomes of young *A. donax* plants subjected to osmotic/water stress with 10 and 20 % polyethylene glycol (PEG; 3 biological replicates/organ/condition corresponding to 18 RNA-Seq libraries), and identified a total of 3034 differentially expressed genes. Blast-based mining of stress-related genes indicated the higher responsivity of roots compared to shoots at the early stages of water stress especially under the milder PEG treatment, with a majority of genes responsive to salt, oxidative, and dehydration stress. Analysis of gene ontology terms underlined the qualitatively different responses between root and shoot tissues. Among the most significantly enriched metabolic pathways identified using a Fisher’s exact test with FDR correction, a crucial role was played in both shoots and roots by genes involved in the signaling cascade of abscisic acid. We further identified relatively large organ-specific differences in the patterns of drought-related transcription factor AP2-EREBP, AUX/IAA, MYB, bZIP, C_2_H_2_, and GRAS families, which may underlie the transcriptional reprogramming differences between organs. Through comparative analyses with major Poaceae species based on Blast, we finally identified a set of 53 orthologs that can be considered as a core of evolutionary conserved genes important to mediate water stress responses in the family.

**Conclusions:**

This study provides the first characterization of *A. donax* transcriptome in response to water stress, thus shedding novel light at the molecular level on the mechanisms of stress response and adaptation in this emerging bioenergy species. The inventory of early-responsive genes to water stress identified could constitute useful markers of the physiological status of *A. donax* and be a basis for the improvement of its productivity under water limitation. The full water-stressed *A. donax* transcriptome is available for Blast-based homology searches through a dedicated web server (http://ecogenomics.fmach.it/arundo/).

**Electronic supplementary material:**

The online version of this article (doi:10.1186/s13068-016-0471-8) contains supplementary material, which is available to authorized users.

## Background

Among the different sources of renewable energy, biomass is interesting because it has a nearly neutral carbon balance and the ethanol produced by its fermentation can be blended with petrol-derived fuels giving an important contribution in reducing transport-related CO_2_ emissions [1]. So-called second generation bioethanol (i.e., the one not produced from edible parts of crops) can be obtained from food crop straw, but the yields of such biomass source are expected to be low, as food crops were intentionally selected to maximize photosynthate allocation to edible parts [2]. In alternative, plant species specifically dedicated to energy production (called bioenergy crops) are normally better biomass producers than food crops, resulting in higher ethanol yields per unit of cultivated area. *Arundo donax* has been identified among bioenergy crops as the most promising species for the Mediterranean area [3]. *A. donax*, commonly called giant reed, is a perennial C_3_, polyploid, bamboo-like grass of the Poaceae family. It favors well-drained soils with abundant moisture, where it can form dense stands up to 6–10 m high with yields of up to 40 tons per hectare each year (comparable, or even exceeding, those of some C_4_ species) [4]. The origins of the giant reed are still debated, but the latest evidences from plastid DNA sequencing and morphometric parameters data collected from 127 herbarium specimens support a Middle-East origin of *A. donax* [5]. Despite the production of panicle-like flowers, no viable seeds from Mediterranean ecotypes have been reported so far [6]. Natural propagation exclusively occurs vegetatively by rooting of rhizome and stem fragments originating as a consequence of floodings, followed by a slow colonization through rhizome expansion [7]. Consistently, genetic diversity in *A. donax* has been reported to be low, but, possibly due to somatic mutation, detectable [8]. Possibly because of its high ploidy, the low intraspecific diversity of *A. donax* does not seem to be associated to fitness tradeoffs, as indicated by its high resistance to biotic and abiotic stresses [9]. If on one hand this resistance causes the high invasiveness of this plant, on the other hand it makes *A. dona*x an excellent bioenergy crop, which can grow with very low management input (e.g., pesticides, fertilization, irrigation) even in marginal lands or in fields irrigated with waste or salty water [10].

The recent advent of Next Generation Sequencing (NGS) has made the development of genomic resources progressively simpler and cheaper [11]. RNA sequencing (RNA-Seq) is to date by far the most powerful tool for the rapid and inexpensive development of genetic resources for any species of interest. In addition, RNA-Seq allows at once the quantitative determination of the expression levels of virtually all transcribed genes in a specific organ, thus providing an extremely powerful tool for the identification of transcripts differentially expressed in response to the abiotic and biotic stresses which negatively impact crop growth and productivity [12].

It is widely accepted that global warming will increase the duration and frequency of drought periods over the 21st century [13]. Many countries already started to develop mitigation strategies to avoid this major threat, which could potentially offset the productivity gains expected from advances in both agricultural and crop breeding techniques [14]. Drought, the condition resulting from limitation of the water available for normal plant growth and development, is one of the extreme environmental conditions that curtails agricultural crop productivity [15]. The first response of plants to water limitation is usually avoidance, a strategy that aims at maintaining a neutral balance between water gained from the root system and lost by transpiration through the stomata. In case of short-term or relatively mild water stress, avoidance can maintain performance and prevent negative effects on plant growth. From a physiological point of view, this is usually achieved by increasing the osmotic potential of root cells, increasing root growth as well as reducing water loss by modulation of stomatal conductance [16]. These physiological adjustments are the consequence of complex cellular changes like: (1) the reprogramming of the cellular metabolism, which shifts to polysaccharide degradation and aminoacid biosynthesis to allow for the accumulation of solutes with an osmotic function (e.g., glycine–betaine, proline, mannitol, etc.), (2) the production of abscisic acid (ABA, a phytohormone mainly associated to seed dormancy and water stress, which causes a reduction of stomatal conductance through closure of stomata) and other phytohormones, and (3) an increased synthesis of proteins for cellular protection/detoxification (late-embryogenesis-abundant, LEA; chaperones and heat stress-proteins necessary for proper protein folding), (4) extensive modulation of ribosomal activity to support active cell growth and division in the root system [16, 17]. When avoidance strategies are not sufficient alone to prevent the onset of water stress, either because of the excessive length or magnitude of the water deficit, tolerance responses become progressively more relevant to limit the damages caused by the reduced availability of water. The same physiological and molecular changes are, however, often shared between the two types of responses, so that a clear-cut distinction between them is not always possible. The medium to long-term adjustments associated to tolerance encompass the development of, e.g., thicker epicuticular waxes to limit water evaporation through epidermal cells, the further decrease of the shoot/root biomass ratio and the allocation of resources to long-term survival organs (e.g., tubers or rhizomes), the enhancement of antioxidant capacity to detoxify the reactive oxygen species (ROS) consequent to photosynthetic limitation, the thickening of xylematic cell walls to prevent collapsing of vasculature, etc. [16, 17].

This complex series of cellular responses to water limitation obviously requires also a profound reprogramming of gene expression. Our understanding of the genetic bases of drought resistance largely benefitted from forward genetic screens in model or crop species (e.g., *Arabidopsis thaliana*, rice, maize, wheat, reviewed by [17]). In addition, several studies devoted to the dissection of the transcriptional responses to drought stress or water deficit conditions have been carried out for the most common cereal crop species (e.g., rice [18]; sorghum [19]). More recently also Poaceae species used exclusively or partly as energy crops (e.g., switchgrass [20]; miscanthus [21]) have been the object of transcriptomic studies which could provide a robust comparative basis in poorly characterized species like *A. donax*. Unfortunately, the high spatio-temporal complexity of the physiological adaptations to drought and the large number of variables used in different experimental protocols for the application of water stress (methods for induction of water deprivation, combination with other stresses, length of treatment, type of plant materials and their developmental stages) limit comparability among studies [22]. Polyethylene glycol (PEG) is a high-molecular weight polymer which can be used to induce controlled water deficits in plants by modifying the osmotic potential of water in hydroponic growth media without being absorbed by the root system [23], thus providing an ideal method for water deprivation in RNA-Seq experiments addressing short-term responses of plants to water stress.

*Arundo donax* is one of the most promising biomass resources for biofuel development but, up to now, little is known at the molecular level on this species’ ability to cope with abiotic stresses in general and in particular with water limitation. Leveraging on the recent obtainment of the first reference transcriptome of *A. donax* by RNA-Seq [24] and on the existing knowledge of the genetics of drought responses in plants, in this study we report the characterization of early transcriptional responses to two levels of PEG-induced water deficit in cohorts of young giant reed cuttings. In particular, we addressed the main questions: (1) How many/which genes are differentially expressed during the early phases of water stress in *A. donax*? (2) What are the main biological functions involved? (3) Which are the transcription factors associated to such transcriptional reprogramming? (4) Are the transcriptional responses of *A. donax* conserved/comparable to those of other monocot species, and in particular of rice?

The set of about 3000 early-responsive genes to water stress identified in this study are promising reporters of the physiological status of *A. donax* plantations for the improvement of its management and for a deeper understanding of its biology.

## Results and discussion

Despite the ability of *A. donax* to withstand prolonged periods of drought, its productivity under water limitation is negatively affected [3]. In this study, we carried out by RNA-Seq a comprehensive identification of early transcriptional responses of *A. donax* shoots and roots to two different levels of PEG-induced water limitation (see experimental design in “Methods” section and in Additional file 1: Table S1).

Following assembly, we obtained 111,749 transcripts covering 45,821 components. Given the high ploidy of *A. donax*, we chose to use a relatively high Kmer coverage during assembly (min-kmer_cov = 5) to minimize the formation of transcripts with retained introns [25]. The observed N50 of the assembled transcriptome is 1826 bp, in line with our previous N50 reports [24], indicating that a good coverage of the transcriptome has been achieved. To eliminate redundant transcripts, we further clustered the transcripts using the CD-HIT software resulting in a total of 80,962 transcripts. The non-redundant transcript set was further assembled into unigenes with MIRA to remove spurious transcripts, resulting in a final set of 80,335 unigenes with an N50 of 1570 bp. Summary statistics results for transcriptome assembly are provided in Table 1.

**Table 1:** Summary statistics of the sequencing reads and the corresponding assemblies

Assembly^a^	Summary statistics
Total trinity transcripts	111,749
Total trinity components	45,821
Contig N50 (bp)	1826
MIRA unigenes	80,355
Total length of sequence (bp)	75,960,964
Unigene N50 (bp)	1570
GC %	47.70

Summary statistics of *A. donax* whole drought transcriptome obtained by Trinity and MIRA.

^a^Trinity assembly: K 25, Kmer coverage 5

### Identification of differentially expressed genes (DEGs) by RNA-Seq

As a first step in the characterization of *A. donax* transcriptional responses to water stress, we carried out the identification of the unigenes whose expression level significantly changed upon PEG-treatments. A total of 3034 genes showed differential expression in at least one of the two stress conditions (mild water stress vs. control, severe stress vs. control and severe vs. mild stress), with roughly the same number of genes being differentially expressed in shoots and roots (1684 and 1712 DEGs, respectively). Validation of expression levels for ten selected DEG candidates was carried out by real-time qRT-PCR, (Additional file 2: Table S2). The high congruence between RNA-Seq and real-time PCR results (coefficient of determination *R*^2^ = 0.94) indicates the reliability of RNA-Seq quantification of gene expression. Therefore, the selected genes could also constitute useful markers of early water deficit in *A. donax.*

DEG identified in biological replicates clustered together in both organs, indicating good reproducibility of treatments. In addition, the heat maps qualitatively indicated the closer similarity of control and mild water stress between each other as compared to severe water stress (Additional file 3: Figure S1).

**Fig. 1:**
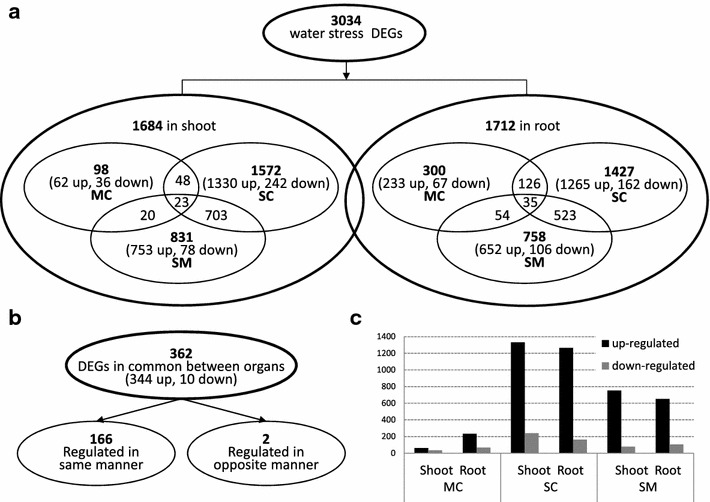
Summary of DEGs in shoots and roots of *A. donax* upon drought stress. **a** Number of genes up-/down-regulated by drought stress under different conditions (*MC* mild water stress vs. control, *SC* severe water stress vs. control, *SM* severe water stress vs. mild water stress.) in root and shoot. **b** Total number of DEGs in common between root and shoot. **c** Number of regulated genes between different conditions. *Gray bar* down-regulated genes; *black bar* up-regulated genes

A detailed assessment of the number and the identity of the DEGs between conditions for each organ confirmed this observation: in shoots, only 98 genes were differentially expressed between control condition and mild water stress, *versus* 1572 between control and severe water stress, and 831 between mild and severe water stress. A similar trend, but less marked, characterized also root DEGs (Fig. 1a), indicating the successful induction of varying degrees of water stress as a function of PEG concentration [23]. By comparing the 362 DEGs in common between organs, we further observed a general conservation of expression patterns, with 166 of the genes being regulated in the same way in shoot and root and only two genes displaying opposite regulation (Fig. 1b). Also the overall direction of expression variation resulted to be conserved between organs, with the large majority of DEGs being up—rather than down-regulated (Fig. 1c). A closer analysis of the absolute numbers of DEGs in the two organs, however, highlighted a relatively large difference in gene up-regulation upon mild stress in roots as compared to shoots (300 DEGs in root vs. 98 in shoot; Fig. 1a). Given the application of the PEG directly to the root system and the sampling of only one time point, it is possible that, at least in part, these differences could stem from a faster onset of the water stress in roots compared to shoots. These results, however, are also in line with a transcriptionally higher responsivity of the root system compared to shoots, as previously reported, e.g., in the case of poplar [26], which could indicate tissue-specific responses.

### Functional classification of transcriptional responses to water stress in *A. donax*

**Fig. 2:**
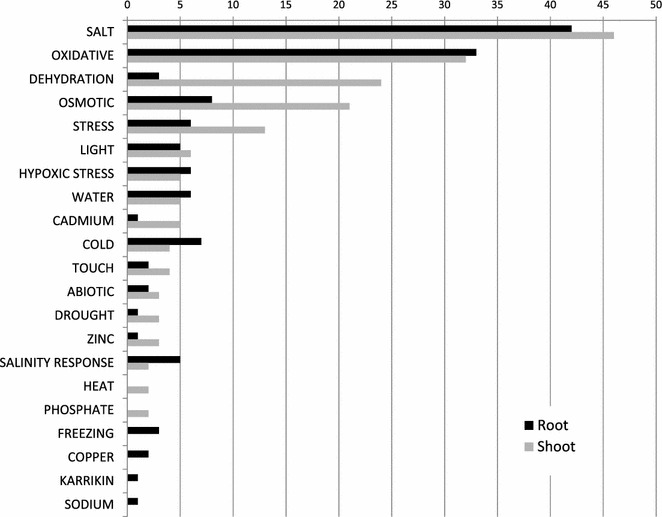
Distribution of stress-related functional categories of DEGs. Stress-related functional categories are identified by annotation of *A. donax* putative homologs in of *Arabidopsis* genes from ASPRGDB. Data are sorted by number of shoot DEGs. *Black bar* root DEGs; *gray bar* shoot DEGs

To identify organ-specific differences, stress-related genes were identified based on curated homology searches against genes experimentally characterized in previous studies. The majority of stress-related genes belonged to categories “salt,” “oxidative,” “dehydration,” and “osmotic.” This is expected, as water limitation is known to cause reduced turgor and integrity of membranes, increase of intracellular ionic and non-ionic solute concentrations and enhanced production of reactive oxygen species (ROS) that cross-trigger responses to high-salinity, oxidative and osmotic stresses [27] (Fig. 2; Additional file 4: Table S3). Worth of note, the two differentially expressed categories encompassing the largest differences in number of genes between organs are “dehydration” and “osmotic.” Both categories are more abundant in shoot than root, but the highest shoot/root ratio (eight times) is found for dehydration-related genes (Fig. 2).

We next carried out a homology-based annotation specifically for all the 3034 DEGs identified upon PEG treatments, identifying at the same time the GO terms associated to this dataset. (Additional file 5: Table S4). Based on BLASTN searches using a 70 % identity and 50 % query coverage cutoff, we identified a total of 214 differentially expressed *A. donax* unigenes in shoots and 642 in roots, which were not present in the reference transcriptome assembly (Additional file 4: Table S3;) [24], thus contributing to ongoing gene discovery and functional annotation in this poorly characterized species.

**Fig. 3:**
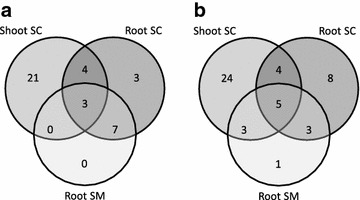
*Venn diagram* of significantly enriched GOs. The GO terms which were overrepresented under different conditions have been slimmed by REVIGO, and compared by category: **a** molecular function; **b** biological process. *MC* mild water stress vs. control, *SC* severe water stress *vs* control; *SM* severe water stress vs. mild water stress

To determine the gene functional classes which were chiefly involved in the response to water stress, we carried out an analysis of over/under-representation of GO terms associated to DEGs. A total of three contrasts were carried out: mild water stress vs. control (MC), severe water stress vs. control (SC) and severe water stress vs. mild water stress (SM). We further selected the most significantly enriched GOs using REVIGO [28] (Additional file 6: Table S5) and analyzed the number of GO terms in common between contrasts to pinpoint differences and similarities between organs and conditions (Fig. 3: shoot/SC; root/SC; root/SM for both (1) molecular function and (2) biological process terms).

The seven molecular function and nine biological process GOs consistently over-represented in SC shoot and root contrasts, respectively (see Additional file 6: Tables S5; Fig. 3), delineated an ongoing reprogramming of cellular transcription and post-translational protein modification related to drought recovery and osmotic adjustment in both organs, in line with the severity of the water stress applied [16]. In particular, the changes observed in proline metabolism in plants (GO:0004657 proline dehydrogenase activity, GO:0006562 proline catabolic process) are a well-known response to a multiplicity of abiotic stresses, including drought [29]. The majority of GO terms were, however, specific for shoot and root and the transcriptional response in the shoot involved 2–3 times more functions than in root (Fig. 3), indicating either different kinetics of water stress onset in the experimental conditions used or qualitative and quantitative differences in the responses of these organs to water deprivation in root several DEGs were associated to functions related to polysaccharide catabolism (e.g., GO:0000272, polysaccharide catabolic process; GO:0016161, beta-amylase activity), indicating extensive osmotic adjustment to reduce the water potential and limit cellular damage [16]. The enrichment of terms related to biotic stress (e.g., GO:0009816, defense response to bacterium incompatible interaction; GO:0009607, response to biotic stimulus) has been reported, e.g., in sorghum tissue treated with PEG or ABA [29], thus supporting the conservation of the cross talk between biotic and abiotic stress responses in Poaceae. Also enrichment of gene functions related to translation was observed (e.g., GO:0003735, structural constituent of ribosome; GO:0006412, translation), possibly as a response to the extensive transcriptional reprogramming observed in roots and/or to root cell growth and division. The extension of the root apparatus is indeed a common response in plants to water stress which maximizes the chance of reaching the moisture available in deeper layers of soil [16]. Worth of note, in root the only biological function specific to the milder PEG treatment (SM contrast, GO:0009685gibberellin metabolic process; Fig. 3b) indicates a possible involvement of gibberellins (GA) in the control of this trait through root growth. Based on the comparison of emmer wheat susceptible and resistant varieties, GA signaling and biosynthesis genes have been associated to resistance to drought in roots [30]. These results are consistent with a role of GA in the maintenance of root growth as part of the developmental decrease of the shoot/root biomass ratio usually observed in plants growing under water stress [31]. It is thus possible that the enrichment of functions related to GA observed also in *A. donax* could contribute to the onset of the developmental changes triggered by mild water stress to increase accessibility of roots to soil with higher moisture.

Compared to root, in shoot the pattern of GO terms enrichment in response to water limitation was dominated by functions related to signal transduction and protein modification associated to phosphorylation (Additional file 6: Tables S5; Fig. 3). This result mirrors the dramatic increase in post-translational phosphorylation levels observed in wheat leaves under drought stress [32]. Interestingly, several other functional classes specifically enriched in *A. donax* shoot transcriptome corresponded to those of proteins undergoing phosphorylation in wheat (e.g., GO:0009405pathogenesis, GO:0008643carbohydrate transport, GO:0005509calcium ion binding, GO:0015291secondary active transmembrane transporter activity), indicating that a synergistic effect between transcriptional and post-translation reprogramming may take place in Poaceae shoots during water stress [32].

Taken together, the identified DEGs indicate major differences between organs in the transcriptional responses to water stress: Roots experienced a seemingly more severe/earlier stress, whereas in shoots the transcriptional response was still mainly at the level of signal transduction. Time course analyses will be required to precisely define the relative contribution of stress induction kinetics *versus* organ-specificity to the patterns of differential expression observed in this study. Given the relevance that the root system plays in both acclimation and adaptation of plants to water stress [33], several of the early-responsive genes identified could constitute suitable markers for the detection of early water stress in *A. donax*.

### Metabolic pathways related to water stress in *A. donax*

**Fig. 4:**
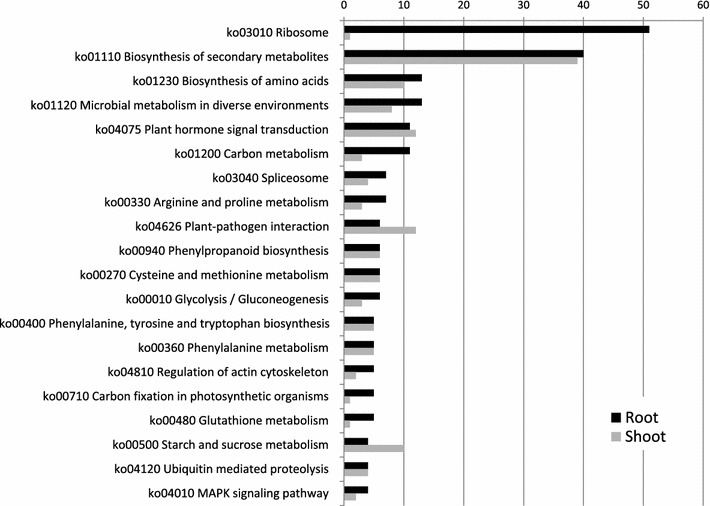
Distribution KEGG Pathways for DEGs in shoot and root. Data are sorted by number of root DEGs mapping to KEGG pathways. *Black bar* root DEGs; *gray bar* shoot DEGs

The set of 3034 DEGs was mapped onto KEGG pathways in *Arabidopsis thaliana* and *Oryza sativa*, highlighting the involvement of several drought-related pathways (Fig. 4).

‘Plant hormone signal transduction’ (ko04075), comprising 11 DEGs in roots and 12 DEGs in shoots, was overrepresented. In this pathway, for both shoots and roots, the transcripts of several hormone-responsive proteins involved in regulation and signal transduction were up-regulated. Plant hormones play crucial roles in a diverse set of developmental processes, as well as in the response to biotic and abiotic stresses [34]. For example, MYC2 is known to function as an activator in ABA signaling and its overexpression in *Arabidopsis* confers increased tolerance to drought [35]. As discussed in more detail below, also ABA-activated SnRK2 is required for dehydration stress signaling in *Arabidopsis* [36]. Previous studies also suggested that in rice OsJAZ1 could connect the jasmonate and drought stress signaling cascades by functionally interacting with OsbHLH148 and OsCOI1 [37]. Taken together these results confirm the pivotal role played in water stress response by the differential regulation of genes involved in hormone signal transduction [27].

Two other important pathways, including ‘Phenylalanine metabolism’ (ko00360) and ‘Plant-pathogen interaction’ (ko04626), were also found in our study to be regulated by water stress (Fig. 4). *CYP73A* (*trans*-*cinnamate 4*-*monooxygenase*), *4*-*coumarate*–*CoA ligase* and *peroxidase*, associated with ‘phenylalanine metabolism,’ were all highly accumulated in response to water stress, in agreement to the relevance of this pathway in plant responses to drought [38]. Additionally, several transcripts from CALM (calmodulin), CML (calcium-binding protein: CaM-like protein), MYC2, RBOH (respiratory burst oxidase), PR1 (pathogenesis-related protein 1) and JAZ members of the ‘Plant-pathogen interaction’ pathway (e.g., CALM, CML, MYC2, RBOH, PR1, and JAZ members) were also induced by water stress. All these genes have been reported to be involved in response to several stresses. For example, as calcium is one of the most important signaling molecules in plants, the expression of CALM and CMLs is well regulated due to different environmental requirements in Arabidopsis [39]. Finally, *RBOH* genes are also commonly expressed in many plants in response to biotic and abiotic stresses [40].

Other examples of relevant pathways which are known to be involved in responses to abiotic stresses in general or specifically to drought were ‘Starch and sucrose metabolism’ (ko00500), ‘Arginine and proline metabolism’ (ko00330), and ‘MAPK signaling pathway’ (ko04010) [27, 41, 42],

Strikingly, the biggest difference observed between root and shoot was related to ribosomal DEGs (ko03010 ribosome; Fig. 4), thus identifying reprogramming of ribosomal translation as one of the largest responses of the root system during the early stages of water stress in *A. donax*. As noted above, it is likely that such large effect to translation could represent the early phases of the modulation of shoot/root resource allocation preluding to root cell growth and division, a typical avoidance responses of the root system during the early phases of water stress. Given the high number of ribosomal subunit genes and the complexity of their regulation as a function of water stress intensity/duration as well as species- and even genotype-dependent variation [43], the detailed dissection of ribosome-related pathway reprogramming will be relevant for the elucidation of root-specific responses to early water stress in *A. donax*.

### Identification of transcription factors responsive to water stress in *A. donax*

Transcription factors have been identified among the most promising targets for the improvement of plant performance under drought stress. Mining of DEGs for putative TFs and their interactors led to the identification of 238 *A. donax* unigenes, corresponding to 136 high confidence rice homologs previously identified as drought-responsive genes from 37 TF families (Additional file 7: Table S6) [44]. Because of the altered water potential under salt stress [27], the majority of these genes (108) are also responsive to salinity. A total of 18 genes, are, however, specifically responding to drought (Additional file 7: Table S6). The most represented *A. donax* differentially expressed families, constituting alone the majority of the genes, were those of NAC, WRKY, AP2-EREBP, bHLH, bZIP, and AUX/IAA, which are known to mediate water stress responses in plants [45]. The majority of these families were also among the most represented in drought-stressed rice [18]. *A. donax* unigenes from the NAC family are the most common among differentially expressed TF genes (36 in total), matching a total of 14 different rice loci. Six of them (Os03g60080/SNAC1; Os01g66120/SNAC2/OsNAC6; Os11g08210/OsNAC5; Os11g03300/OsNAC10; Os08g06140; Os05g34830) have been previously identified as drought responsive [46]. Four of them have been characterized in depth through functional analyses, confirming their pivotal role in water stress-related transcriptional reprogramming in rice. In particular, all of them have been demonstrated to be ABA-responsive [47–50], in agreement with the activation of the ABA signal translation cascade observed above.

**Fig. 5:**
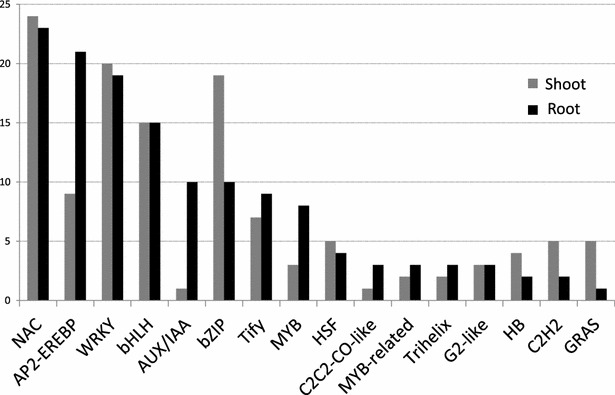
Distribution of transcription factors responsive to water stress in *A. donax*. Data are sorted by number of root DEGs. Only categories with more than 3 DEGs identified as transcription factors are shown. *Black bar* root DEGs; *gray bar* shoot DEGs

A total of 67 out of the 150 rice homologs to differentially expressed *A. donax* TF unigenes (45 %) were consistently found to be differentially expressed also in rice (Additional file 7: Table S6.) [51]. Not all the TF families, however, were equally represented in both *A. donax* organs, indicating that part of the differences observed between shoot and root transcriptional responses may be mediated by members of these groups. In root among the families encompassing more than five differentially expressed unigenes, we found twice as many AP2-EREBP, AUX/IAA, and MYB unigenes than in shoot. AP2-EREBP is a superfamily of transcription factors composed by the ERF, AP2, and RAV families (Fig. 5). AP2/EREBP TFs are involved in many fundamental biological processes, ranging from development to response to biotic and abiotic stresses [52]. Five of the *A. donax* unigenes, homologous to rice genes Os02g51670 (DREB2B), Os09g20350 (DREBF1), Os04g55520 (DREB2F), and Os06g03670 (OsDREB1C/CBF), belong to the DREB subfamily of ERF TFs, which are known to control expression of several genes in response to dehydration and low temperature [53]. All the four rice homologs have been directly involved in the responses to water stress, thus confirming their relevance towards this stress also in *A. donax* [54–57]. Other *A. donax* unigenes from the AP2/EREBP super-family were homologs of 4 rice ERF genes (Os03g09170; Os08g31580; Os06g10780; Os05g41780). Unlike DREB genes, ERF TFs have been associated mainly to biotic stress responses mediated by ethylene [58]. It is, therefore, likely that the *A.donax* ERF TFs identified in this study are at least in part responsible for the enrichment of GO terms related to biotic stresses observed above, possibly with the participation of members of the NAC and WRKY families [59].

The second TF family with most unigenes in roots compared to shoot was that of AUX/IAA. About ten times more *A. donax* unigenes from this family were differentially expressed in root upon water stress compared to shoot (Fig. 5). AUX-IAA proteins interact with TFs of the ARF (Auxin-Responsive Factors) family, repressing root growth as consequence of the increase of the intracellular levels of the auxin plant hormone indoleacetic acid (IAA) [60]. Since *A. donax* is a perennial plant whose large rhizomes serve as a long-term survival organ, the AUX-IAA genes specifically induced in roots are interesting candidates to dissect the coordination of *A. donax* root and shoot growth under water deprivation.

MYB and MYB-related transcription factors also were more represented in roots as compared to shoots (Fig. 5). Two of the *A. donax* MYB unigenes were homologs to rice Os12g37690, which had been previously associated to differences among drought-sensitive and drought-tolerant rice cultivars [61]. The gene is also upregulated in response to oxidative stress during the early response of japonica rice to chilling [62]. Interestingly, another *A. donax* unigene was homolog to Os12g37970, a rice MYB TF involved in the coordinate regulation of cellulose and lignin biosynthesis [63], indicating that it could contribute to alter the structure of cell walls in response to water stress.

Among the largest TF families displaying a higher number of differentially expressed unigenes in *A. donax* shoots compared to roots we found the bZIP, C_2_H_2_, and GRAS families (Fig. 5). Strikingly, nine out of the 21 differentially expressed bZIP unigenes were homologous to rice Os02g52780. This rice gene, also called OsbZIP23 [64], has been functionally demonstrated to have a relevant role among rice bZIP genes in conferring ABA-dependent drought and salinity tolerance [65]. Somehow unexpectedly, none of the rice genes homologs to differentially expressed *A. donax* unigenes (LOC_Os07g39470, LOC_Os01g62460, LOC_Os01g71970, LOC_Os07g36170, LOC_Os11g47870) have been functionally characterized, leaving open their specific roles in drought responses. Two of them (Os01g71970 and Os07g36170), however, had already been identified among the few GRAS TFs differentially expressed in rice upon drought stress [51]. More recently, additional evidences for the involvement of members of the GRAS family in the responses to water deprivation have been reported for rice [66]. Consistently with our results in *A. donax*, expression of *OsGRAS23* was significantly induced in rice leaves following treatments with PEG, dehydration, salt, gibberellins, and jasmonic acid. In particular, transgenic rice overexpressing *OsGRAS23* (LOC_Os04g50060) was more resistant to drought and tolerant to oxidative stress compared with wild-type plants, thanks to the upregulation of genes involved in antioxidant functions [66]. Taken together, these results indicate that GRAS genes in general and in particular those identified in *A. donax* represent interesting candidates for increasing water stress tolerance in monocots. Also the majority of the rice homologs of differentially expressed *A. donax* unigenes from the C_2_H_2_ family (LOC_Os03g55540, LOC_Os03g13600, LOC_Os03g60570, LOC_Os09g38340) were previously identified as drought responsive [66], indicating their conserved role in Poaceae. Two among them and an additional C_2_H_2_ gene not previously identified (LOC_Os03g10140, LOC_Os09g38340, LOC_Os09g38790) are known to control the vegetative to floral phase transition in monocots [67, 68], indicating that responsiveness of C_2_H_2_ genes to water deprivation may be relictual in *A. donax*: while other species from the *Arundo* genus are fertile and could benefit from accelerating seed setting as a drought-escape strategy, *A. donax* is fully sterile [69] and no clear selective advantage seems to be associated to this trait. Therefore, loss of function mutations of C_2_H_2_ or other flowering time TFs could be interesting candidates to extend the vegetative phase and, thus, biomass accumulation in *A. donax* [24].

### Characterization of co-regulated gene expression network in *A. donax*

We compared the distribution of both differentially and non-differentially expressed *A. donax* genes with the 15 drought-responsive modules of rice orthologs recently identified [70]. Only Module 7 and Module 14 were over-represented in both shoots and roots, while Module 10 was over-represented only in shoots (Table 2).

**Table 2:** Comparison between *A. donax* water stress response genes and rice drought response network

Rice module	Rice genes	Putative orthologs in *A. donax*	Putative orthologs in *A. donax* shoot DEGs (*p* value)	Putative orthologs in *A. donax* root DEGs (*p* value)	Putative module function
Module 1	303	149	2	8	
Module 2	213	155	5	4	
Module 3	141	61	1	0	
Module 4	134	35	2	3	
Module 5	117	71	2	2	
Module 6	90	29	4	0	
Module 7	77	46	22 (4.89E−15)	12 (3.66E−07)	Hormonal signal transduction
Module 8	48	18	0	0	
Module 9	47	16	3	2	
Module 10	47	27	8 (3.22E−4)	0	Post-translational protein modification
Module 11	46	11	0	0	
Module 12	42	11	0	0	
Module 13	38	13	0	0	
Module 14	28	17	6 (5.42E−4)	8 (3.53E−07)	Stomatal closure
Module 15	21	11	1	0	

A total of 56 (in shoot) and 39 (in root) *A. donax* DEGs for which rice orthologs could be identified are mapped onto the 15 co-expression modules previously identified in rice [70]. Significance levels for over- and under-representation as compared to rice (p-value) are provided

There are 1392 rice genes in the 15 modules. Based on Blastp reciprocal best hits method, a total of 56 and 39 differentially expressed genes were identified as putative orthologs of rice genes in shoot and root of *A. donax*, respectively

Module 10 had been identified as a post-translational drought-related signaling/regulation cascade (genes involved in protein amino acid phosphorylation processes), further confirming the results from GO enrichments discussed above. The functions of Module 7 and Module 14, however, were not reported. Based on the functional mining of rice and *A. donax* orthologs in each module (Additional file 8: Table S7), we found that Module 7 might be related to hormonal signal transduction, since these genes are mapped on JAZ, CML, PTC2_3, and ABF, which all belong to the ‘Plant hormone signal transduction’ pathway. The observation that PSY (phytoene synthase), which controls metabolic flux through the pathway supplying carotenoid precursors for ABA biosynthesis, is also part of this module further strengthens the identification of Module 7 as likely ABA-related co-expression module. Additionally, promoters of genes from Module 7 in rice were found to be enriched in S-BOX motif, which is the ABI4 binding site. ABI4 is known to be an important link between ABA hormone and glucose signaling pathway, and it has been proposed that in some species carbohydrate metabolism might be the initial response to drought [71]. Thus, genes belonging to Module 7 likely play a conserved role in Poaceae in the ABA-mediated modulation of carbohydrate metabolism in response to water stress, as observed also in some dicotyledonous species [72].A reliable prediction of Module 14 function(s) is more difficult, as this module comprises *PP2C*, a fundamental trigger in stress-related ABA signaling cascade [73] and raffinose synthase [EC:2.4.1.82], which is involved in the biosynthesis of raffinose, an osmoprotectant associated to drought tolerance [74]. Taken together, these results support the view that Module 14 is likely involved in a plant hormone transduction pathway related to ABA, necessary for the early onset of stomatal closure. Recent physiological analyses indicate that *A. donax* can fix CO_2_ at soil water contents close to wilting point, thanks to its ability to effectively control stomatal regulation in relation to soil water content [75]. The association in our transcriptomics data of Module 7 and 14 to ABA-related pathways confirms and further extends this observation, indicating that in *A. donax* such regulation can be activated as early as 1 h after the onset of PEG-induced water stress and that it may contribute to the high adaptability of this species to resource-poor habitats and marginal soils [3].

Despite the incomplete understanding of the functions of the genes comprised in these clusters, selected members of both Module 7 and 14 could constitute, on one hand, suitable markers to dissect early stress responses and, on the other hand, promising targets to modulate drought tolerance in *A. donax*.

Interestingly, in the afore-mentioned study, only Modules 4, 7, and 14 are significantly associated to rice early responses to drought, where an experimental design similar to ours (3 h treatment, two tissues) has been used [70]. This match supports the conservation of early drought response networks between *A. donax* and rice, two species associated to water-rich environments. A closer examination of the genes belonging to the latter three modules extends the possible conservation of drought-related regulatory networks even further. Among the 53 drought response genes in common among *A. donax*, rice, sorghum, and foxtail (see next paragraph), 13.2 % of genes (7 genes) are from Module 14. Considering that there are only 28 genes in Module 14, much less than in the others, especially this module seems to capture a relevant drought-related mechanism across Poaceae species.

### Identification of a core set of Poaceae genes differentially regulated upon water stress

**Fig. 6:**
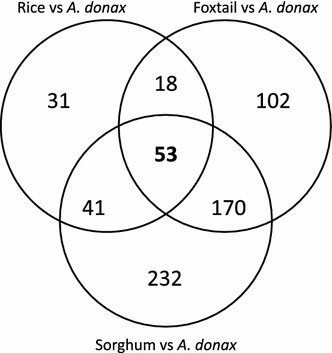
Drought response genes comparison across *A. donax*, rice, foxtail, and sorghum. The Venn diagram represents putative orthologs of *A. donax* stress-responsive genes identified by OrthoMCL in at least two species

The comparison of transcriptomes across different species can provide information about conservation of gene functions over evolutionary time. We, therefore, identified the subsets of water stress-related DEGs in common between *A. donax*, foxtail, sorghum, and rice. When the *A. donax* DEGs were compared with drought-responsive genes reported in previous studies [19, 70, 76], a total of 343, 496, and 143 putative orthologs were identified from foxtail, sorghum, and rice, respectively (Fig. 6; Additional file 9: Table S8). In total 53 groups of putative orthologs present in all species were identified, which constitute a core of evolutionarily conserved genes associated to early responses to water deprivation.

Some of these genes are involved in the quality control and targeted degradation of proteins. For example, a putative ortholog of *Arabidopsis* AT5G51070 gene, which codes for the ClpD subunit of the plastidial Hsp100/Clp complex, a caseinolytic protease (Clp) necessary for chloroplast biogenesis and protein homeostasis (Additional file 9: Table S8) [77]. This gene, also known as *Early Responsive to Dehydration 1* (*ERD1*), is an ATP-dependent molecular chaperone that likely directs unfolded polypeptides to the Clp complex for degradation [78], suggesting that also in *A. donax* it could help eliminate damaged/misfolded proteins and aid proteome reprogramming upon water stress. This observation is supported by the fact that in rice the ClpD protein has been reported to be preferentially upregulated along with several other proteases in response to conditions of active drought signaling but water availability, which in shoots mimicks the early water stress of our study [79].

Several of the other conserved DEGs participate in the biosynthesis of different metabolites, ranging from sugars to hormones, lipids, and flavonoids (Additional file 9: Table S8). Among the most interesting *A. donax* candidates involved in sugar metabolism, there is a putative raffinose synthase protein homolog to *Arabidopsis* AT5G40390, the only isoform reported to be responsive to a wide array of abiotic stresses, including water deficit [80]. Knockout mutants of AT5G40390 have reduced amounts of verbascose, sucrose, and mannitol, but, surprisingly, their shoots are slightly less susceptible to prolonged drought than WT [81]. The *Arundo* homolog of this gene, however, is upregulated in roots, indicating that significant regulatory differences exist between species and suggesting that in the latter species this gene could contribute to short-term osmotic adjustment in roots.

In agreement with previous studies, also a certain number of membrane transporters are among the conserved genes involved in the early response to water stress (Additional file 9: Table S8). For instance, a transporter which was upregulated in both shoots and roots is the homolog of *Arabidopsis* gene AT1G15520, coding for ABCG40, a plasma membrane ABA uptake transporter. In *Arabidopsis*, stomata of *abcg40* mutants respond more slowly to ABA and are less drought tolerant than WT plants [82]. Interestingly, the putative ortholog of AT1G78390, nine-cis-epoxycarotenoid dioxygenase 9, a key enzyme in ABA biosynthesis [83], is strongly upregulated in both *Arundo* shoots and roots during the early responses to water stress. Taken together, these results are in line with the established role of ABA as the main plant hormone in the early responses to water stress [27]. The identification of several differentially expressed ABA-responsive kinases and phosphatases allowed also the definition of a conserved core of signaling genes shared between *Arundo* and the other Poaceae considered. The putative ortholog of *Arabidopsis* AT4G33950 gene is strongly upregulated in water-stressed *Arundo* shoots. This gene is a member of SNF1-related protein kinases (SnRK2) responsive to both ionic and non-ionic osmotic stresses. Among the SnRK2 paralogs, AT4G33950 (also called SnRK2.6) is the most important for overall stomatal control [84]. In *Arabidopsis*, loss of function mutations of this gene completely abolish ABA-mediated stomatal responses, leaving unaffected the ABA-independent reactions and resulting in increased drought susceptibility [85]. Given the proposed involvement of this gene in the early phases of ABA perception before the development of reactive oxygen species associated to cell damage [85], the ortholog of AT4G33950 could constitute an interesting candidate to modulate the responsiveness to water stress responses in *Arundo* and be used as a sensitive marker for shoot drought stress. Two additional kinases are specifically upregulated in *Arundo* leaves subjected to water stress. The first one, homologous to gene AT1G70520, encodes a cysteine-rich receptor-like protein kinase which in *Arabidopsis* has been shown to respond only weakly to ABA and other hormone treatments, but to be upregulated shortly after ozone treatment [86]. The second one is a poorly characterized protein kinase homolog to AT1G56130, one of the four paralogous loci present as tandem duplications in the *Arabidopsis* genome. Possibly due to redundancy, very limited functional information is available about this small gene family. However, the early response and high levels of upregulation in water-stressed shoots of *Arundo* make it an interesting candidate deserving further characterization. In addition to protein kinases, also a phosphatase homologous to *Arabidopsis* AT2G29380 gene, called *Highly ABA*-*Induced1* (*HAI1*), is among the conserved Poaceae DEGs. Like several other water-stress clade A protein phosphatase 2Cs (PP2Cs), *HAI1* acts as a negative regulator of osmoregulatory solute accumulation. Unlike the majority of its closest paralogs, however, the HAI1 protein is largely insensitive to inhibition by members of the ABA receptors family [87]. The concomitant expression of *Arundo* homologs of HAI1 and SnRK2.6 (the latter acting downstream of the other ABA-receptor repressible PP2Cs) [88] raises the interesting possibility that HAI1 may act antagonistically to SnRK2.6 to prevent excessive osmoregulatory solute accumulation. This hypothesis is supported by the fact that *Arabidopsishai1* mutants accumulate higher amounts of proline and other osmoregulatory solutes than wild-type plants [87]. Strikingly, among the conserved early water stress DEGs, the only transcription factor is a homolog of *ATHB7* (Arabidopsis gene AT2G46680), a member of class I plant-specific homeodomain-leucine zipper family [89]. In *Arabidopsis*, *ATHB7* and its paralog *ATHB12* modulate abscisic acid signaling by regulating protein phosphatase 2C and abscisic acid receptor gene activities [90]. Although direct regulation of *HAI1* has not been tested, several other clade A *PP2Cs* are under positive transcriptional regulation by *ATHB7*/*ATHB12*, which at the same time repress transcription of genes from the PYR/PYL family of ABA receptors [90]. As, both in *Arabidopsis* and rice, paralogs with different tissue-specific and developmental expression patterns have been implicated in different aspects of ABA-mediated growth responses to water stress [91], the characterization of *Arundo*’s homeodomain-leucine zipper family members seems to be a promising starting point to dissect the details of abscisic acid signaling modulation and stomatal control in this species.

## Conclusion

The lack of information available about the molecular mechanisms involved in stress responses in *A. donax* is currently a major constraint for the further development of this semi-wild species into a fully fledged bioenergy crop. To fill at least in part this gap, we hereby provided the first characterization of *A. donax* shoot and root transcriptomes in response to water stress, one of the factors of highest concern for its productivity. Given the commonality of the responses to water limitation and other stresses, in addition to providing a general overview of the early transcriptional responses to simulated drought, our results shed also light at the molecular level on the general mechanisms of stress response and adaptation in *A. donax*. Upon functional validation, thus, many of the unigenes identified in the present study have the potential to be used for the development of novel *A. donax* varieties with improved productivity and stress tolerance. In addition, the inventory of early-responsive genes to water stress provided in this study could constitute useful markers of the physiological status of *A. donax* plantations to deepen our understanding of its biology and productivity under water limitation.

## Methods

### Plant material and application of water limitation stress

In the present study, we applied a water stress by treating cohorts of *A. donax* cuttings (collected in Sesto Fiorentino, Florence, Italy 43°49′01.8″N 11°11′57.0″E) with two different concentrations of polyethylene glycol 6000 (PEG; 10 and 20 % w/w, referred to as mild and severe water stress conditions, corresponding to osmotic potentials of −1.54 and −5.04 bars, respectively). Briefly, *A. donax* cuttings were let rooting in tap water, then transferred to 1 % Hoagland solution and grown in a growth chamber with day-length of 16 h, light intensity of 150 µmol of photons m^−2^ s^−1^, 24 °C and 60 % RH. At the five-leaves stage, two cohorts of cuttings were transferred to 1 % Hoagland solution containing either 10 or 20 % PEG, while a third cohort used as control was transferred to 1 % Hoagland solution without PEG. After 1 h shoots (encompassing the three upper leaves; see Additional file 1: Table S1) and whole roots were separately collected from each cohort, quickly rinsed in distilled water and snap-frozen in liquid nitrogen. A total of 18 samples (three biological replicates from both shoot and root for each of the three conditions) were sampled.

### Next generation RNA sequencing

RNA preparation was carried out as described in [35]. Paired-end RNA-Seq libraries were prepared using the TruSeq RNA Sample Prep V2 kit (Illumina, San Diego, CA), pooled in equimolar ratio and sequenced on an Illumina HiSeq 2000 sequencer (CIBIO NGS Facility, Povo (TN), Italy).

A minimum of 694 million reads were obtained from each of the 18 libraries sequenced (Additional file 1: Table S1). RNA-Seq data are available in the ArrayExpress database (www.ebi.ac.uk/arrayexpress) under accession number [ArrayExpress:E-MTAB-3769]. Assessment of read quality metrics was carried out using the FastQC software (available at http://www.bioinformatics.babraham.ac.uk/projects/fastqc/) after which stringent quality filtering, removal of reads containing Ns and de-duplication was carried out as previously described [24].

For transcript reconstruction, we concatenated all read pairs passing the quality checks and assembled them using the Trinity software with-group-pair-distance = 500 and −min-cov = 5 to limit intron retention in reconstructed transcripts [92]. After discarding transcripts shorter than 200 bp, transcript redundancy was reduced using CD-HIT-EST with 95 % identity and a word size of 8 [93]. The resulting non-redundant transcript dataset was further assembled into unigenes using the Overlap-Layout-Consensus assembler MIRA (parameters: job = denovo, est, accurate, 454 using the notraceinfo option) [94]. All final *A. donax* unigenes are available for homology searches and download through a dedicated web Blast server (http://ecogenomics.fmach.it/arundo/) [95].

### Water stress transcriptome annotation

Following the assembly, transcriptome curation was carried out by performing BLASTx searches with *E* value threshold 1E^−5^ against NCBI non-redundant (www.ncbi.nlm.nih.gov), UniProt (www.uniprot.org) and TrEMBL [96] plant databases. Additionally, we also curated the unigenes using the FastAnnotator program [97]. We further slimmed the obtained Gene Ontology (GO) categories using the PlantGO Slim categories available from the Gene Ontology consortium (www.geneontology.org). Protein domains of transcriptome unigenes were identified using InterPro (https://www.ebi.ac.uk/interpro/). To identify the putative homologs of stress-responsive genes characterized so far in *Arabidopsis thaliana*, BLASTx searches were performed with an *E* value threshold of 1E^−5^ against the ASPRGDB database [98], retaining only hits with query sequence coverage and identity higher than 50 %. Additionally, we mined functionally relevant genes involved in drought stress by creating a customized, manually curated database from *Sorghum bicolor*, *Zea mays*, *Arabidopsis thaliana*, and *Oryza sativa*.

### Identification and functional classification of differentially expressed genes

To identify genes which are differentially expressed upon water stress, reads from each of the 18 libraries were individually mapped on the unigene assembly and fragments per kb of exon per million fragments mapped (FPKM) values were estimated as a measure of the expression using RSEM [99]. For the identification of differentially expressed genes, we used EdgeR (R version: 3.0.1, edgeR version: 3.4.2) [100], implementing the Generalized Linear Model (GLM) approach. For the normalization of the read count, we applied the trimmed mean of *M* values (TMM) normalization method. Additionally, contrasts were made to identify the set of genes differentially expressed among control and water stress treatments. A false discovery rate (FDR) cutoff of 0.001 and a log-fold change (LogFC) threshold of 2 were implemented to filter the significantly up- and down-regulated genes between the treatment and the control. The genes with logFC ≥ 2 and logFC ≤ −2 with a FDR cutoff of FDR 0.001 between two treatment conditions were determined to be up-regulated and down-regulated, respectively. All the differentially expressed genes which were significantly up- and down-regulated were custom annotated against the functionally identified drought-responsive genes in model grass species. For the identification of transcription factors responsive to water stress in *A.donax*, we mined the stress-responsive transcription factor database of rice (SRTFDB [44] by Blastn searches with an *E* value cutoff of 1e−5. For the identification of the subsets of water stress-related DEGs in common among *A. donax*, foxtail, sorghum, and rice, OrthoMCL software V5 was used with default settings. [101]. For the comparative study between *A. donax* and rice co-regulation network, we identified putative orthologs between the *A. donax* and rice with Blastp Reciprocal Best hits method (RBH, *E* value 1e^−6^).

### GO enrichment

Blast2GO was also used for a GO functional enrichment analysis of genes, by performing Fisher’s exact test with a robust FDR (<0.05) correction to obtain an adjusted *p* value between each test gene group and the whole transcriptome annotation. We uploaded in REVIGO [28] the lists of over-represented GO IDs along with the *p* value from the result of the fisher’s exact test. The analysis was run by selecting the small size of the resulting list, with the numbers associated to GO categories *p* values, with the *Oryza sativa* database, and the SimRel as the semantic similarity measure (Additional file 6: Table S5). The number of over-represented terms in common between conditions was displayed as Venn diagram.

### Pathway enrichment

To identify functionally relevant patterns associated to water stress in shoot and root DEGs, we created a unigene subset for each organ selecting genes with FPKM ≥ 1. Each subset was subsequently used as background to identify over- and under-represented GO categories among DEGs using the Fisher’s test with a *p* value cut-off of 0.05. In addition, pathway enrichment analysis of DEGs was carried out with the KOBAS software [102] using BLASTx searches against the *Oryza sativa* var. *japonica* proteins.

### Real-time validation of selected DEG candidates using qRT-PCR

Each RNA sample was treated with DNase I (Sigma-Aldrich) and 1 µg of total RNA was reversed transcribed using the SuperScript^®^ III Reverse Transcriptase (Life Technologies), according to the manufacturer’s instructions. Real-time qRT-PCR was performed for a total of 10 DEGs with Platinum^®^ SYBR^®^ Green qPCR SuperMix-UDG and carried out in the Bio-Rad C1000 Thermal Cycler detection system according to the manufacturer’s instructions. All the genes were normalized with putative *A. donax* actin protein with highest homology to sorghum *AC1* gene (GenBank accession no. P53504). Each PCR reaction (12.5 μL) contained 11 μL real-time PCR Mix, 0.25 μM of each primer and 1 µL of a 1:5 dilution of cDNA. The thermal cycling conditions were 50 °C for 2 min, 95 °C for 2 min, followed by 40 cycles of 15 s at 95 °C and 30 s at 60 °C. All reactions were performed in triplicate and fold change measurements calculated with the 2^−ΔΔCT^ method. Sequences of primers used for real-time PCR are provided in Additional file 2: Table S2.

## Additional files

10.1186/s13068-016-0471-8 Experimental design, number of reads per library and sampling scheme.
10.1186/s13068-016-0471-8 Validation of 10 *A. donax* DEGs by Real Time qrt-PCR.
10.1186/s13068-016-0471-8 Visualization of differentially expressed genes. Heat maps visualization of the differentially expressed genes between three conditions for shoots (a) and roots (b) using Euclidean distances between TMM normalized expression values. Expression levels for genes in each cDNA library were measured as fragments per kilobase per million reads (FPKM), and color-coded from green (lowly expressed) to red (highly expressed). Hierarchical clustering level at individual libraries is represented by the dendrogram for shoots (c) and roots (d), and color-coded from green (weak correlation) to red (strong correlation).
10.1186/s13068-016-0471-8Stress-related A. donax DEGs.
10.1186/s13068-016-0471-8 Functional annotation, fold change values and statistical significance of DEGs.
10.1186/s13068-016-0471-8 Slimmed GO terms overrepresented under different conditions.
10.1186/s13068-016-0471-8 Transcription factors responsive to water stress in *A. donax*.
10.1186/s13068-016-0471-8 Comparison of co-regulated expression modules responsive to water stress in *A. donax* and rice.
10.1186/s13068-016-0471-8 Drought responsive orthologs identified among *A. donax*, foxtail, sorghum and rice.

## Abbreviations

DEG: differentially expressed gene
MC: mild water stress vs. control
SC: severe water stress vs. control
SM: severe water stress vs. mild water stress
GHG: green house gas
IPCC: intergovernmental panel on climate change
RAPD: random amplified polymorphic DNA
AFLP: amplified fragment length polymorphism
PCR: polymerase chain reaction
qRT-PCR: quantitative reverse transcription PCR
ISSR: inter-simple sequence repeat
SSR: simple sequence repeat
PEG: polyethylene glycol
FDR: false discovery rate
logFC: log2-fold change
GO: gene ontology
KEGG: kyoto encyclopedia of genes and genomes
FPKM: fragments per kilobase of exon per million fragments mapped
RMBT: reads mapping back to the assembled transcriptome
GLM: generalized Linear Model
TMM: trimmed mean of *M* values
RBH: reciprocal best-hits
ASPRGDB: *Arabidopsis thaliana* stress-responsive gene database
TrEMBL: translated EMBL nucleotide sequence data library
