# Supplementary material for: Dissection of early transcriptional responses to water stress in Arundo donax L. by unigene-based RNA-seq
Source: Biotechnol Biofuels. 2016 Mar 8;9:54. doi: 10.1186/s13068-016-0471-8 (PMC4782572; doi:10.1186/s13068-016-0471-8)
Supplement: Supplementary file 3 — 10.1186/s13068-016-0471-8 Visualization of differentially expressed genes. Heat maps visualization of the differentially expressed genes between three conditions for shoots (a) and roots (b) using Euclidean distances between TMM normalized expression values. Expression levels for genes in each cDNA library were measured as fragments per kilobase per million reads (FPKM), and color-coded from green (lowly expressed) to red (highly expressed). Hierarchical clustering level at individual libraries is represented by the dendrogram for shoots (c) and roots (d), and color-coded from green (weak correlation) to red (strong correlation). [file 13068_2016_471_MOESM3_ESM.pdf]

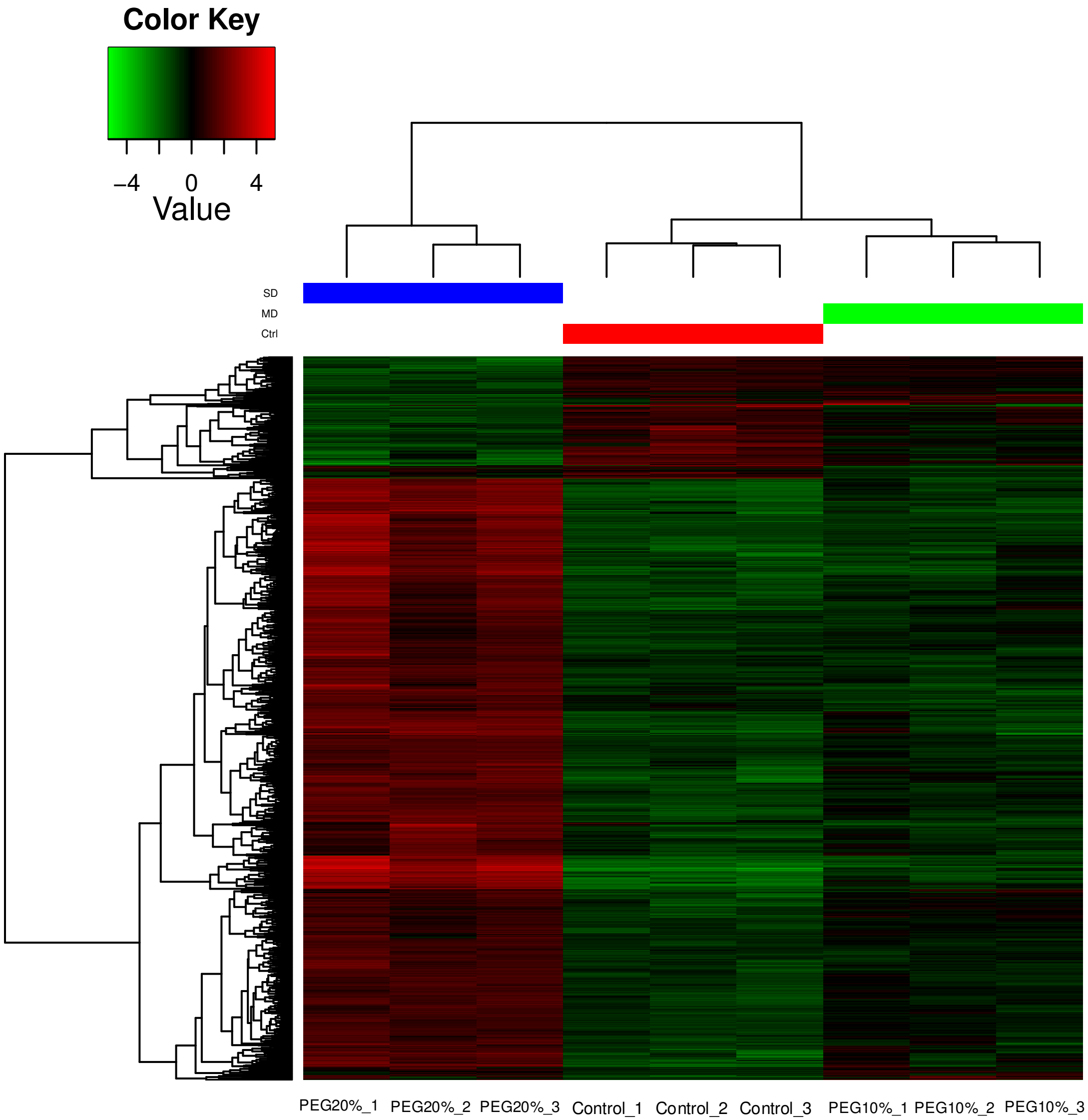
Color Key
−4
0
4
Value
SD
MD
Ctrl
PEG20%_1
PEG20%_2
PEG20%_3
Control_1
Control_2
Control_3
PEG10%_1
PEG10%_2
PEG10%_3

(a)

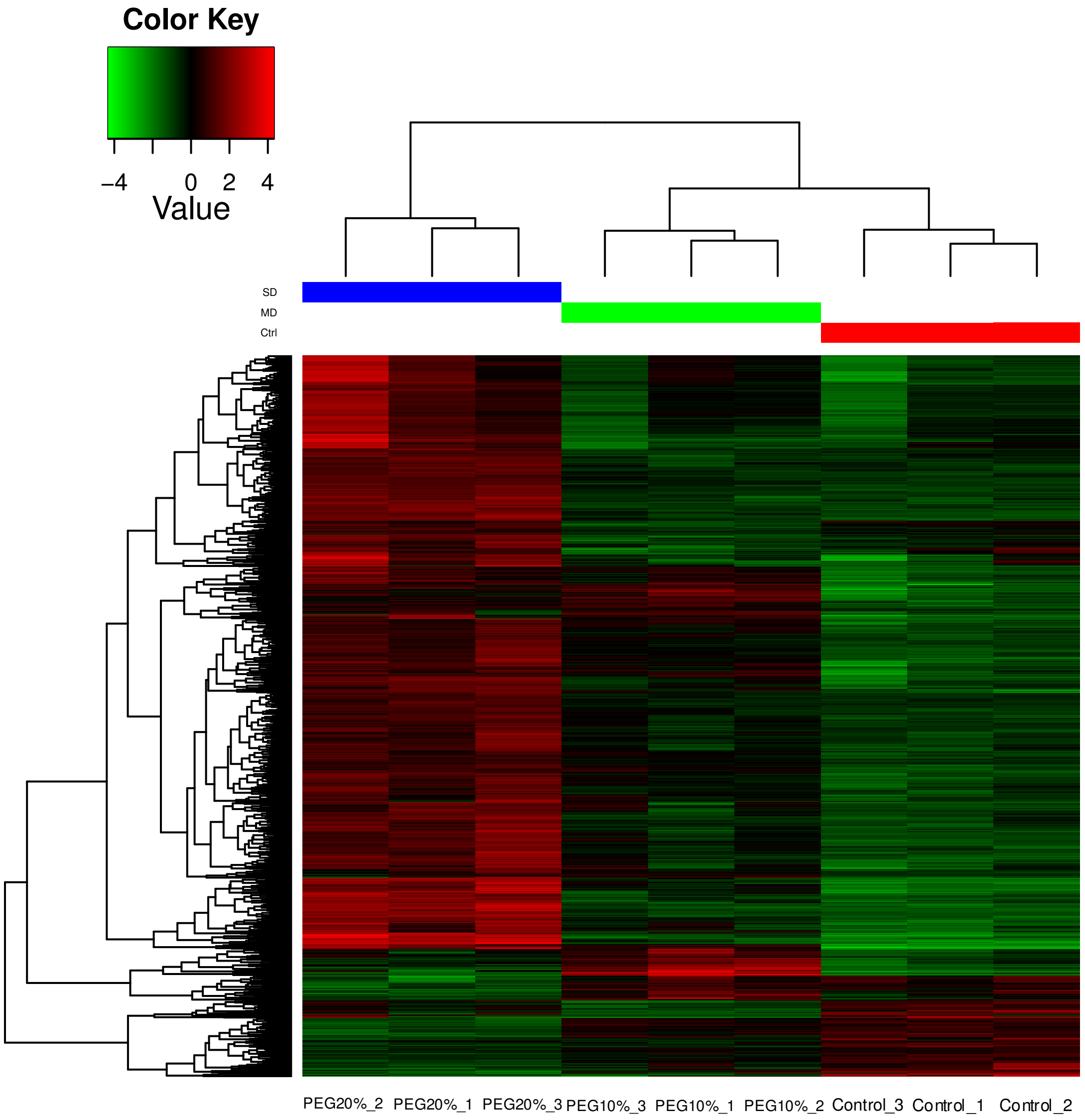

Color Key
−4
0
2
4
Value
SD
MD
Ctrl
PEG20%_2
PEG20%_1
PEG20%_3
PEG10%_3
PEG10%_1
PEG10%_2
Control_3
Control_1
Control_2

(b)

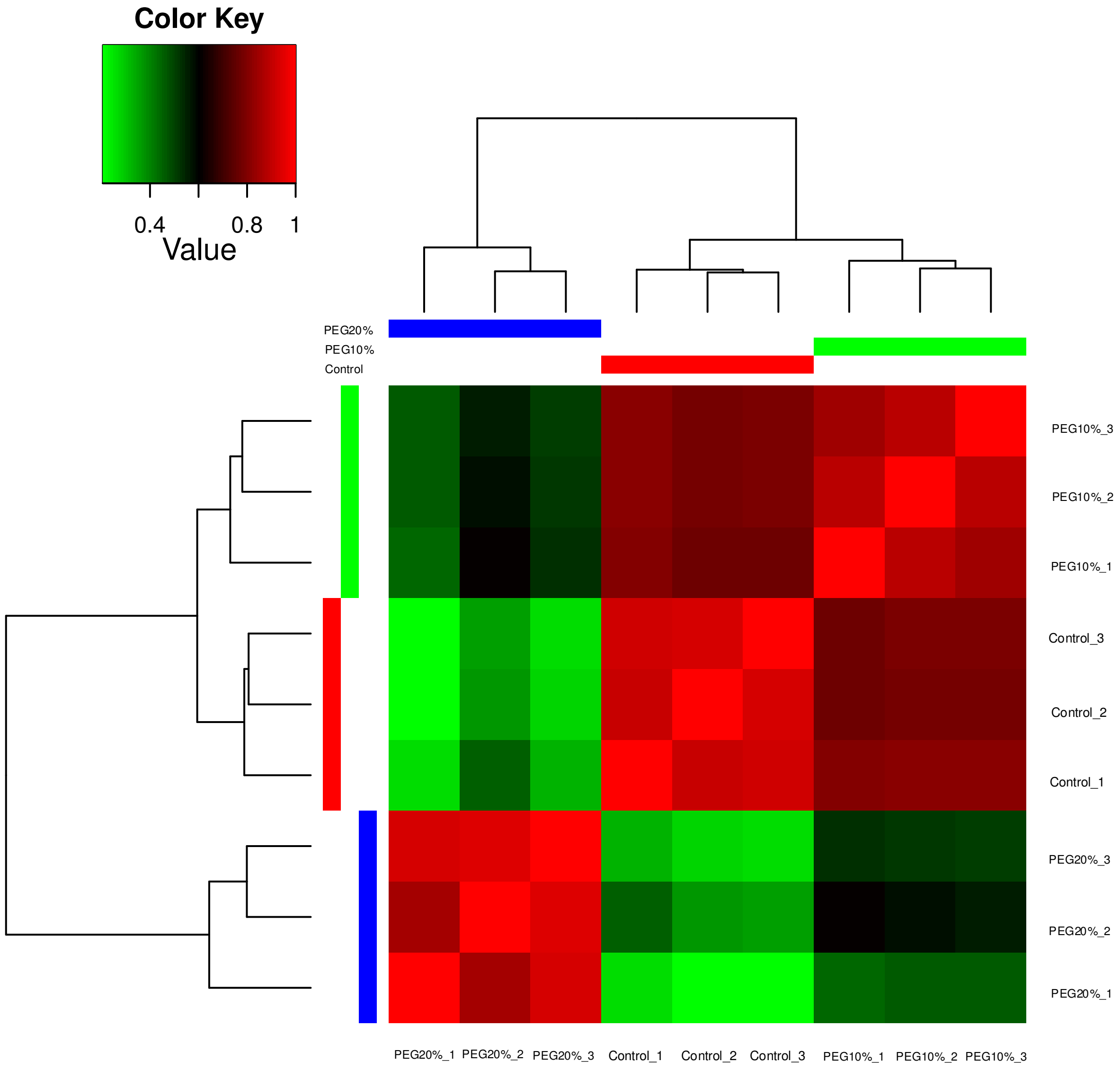
Color Key
0.4
0.8
1
Value
PEG20%
PEG10%
Control
PEG10%_3
PEG10%_2
PEG10%_1
Control_3
Control_2
Control_1
PEG20%_3
PEG20%_2
PEG20%_1
PEG20%_1
PEG20%_2
PEG20%_3
Control_1
Control_2
Control_3
PEG10%_1
PEG10%_2
PEG10%_3

(c)

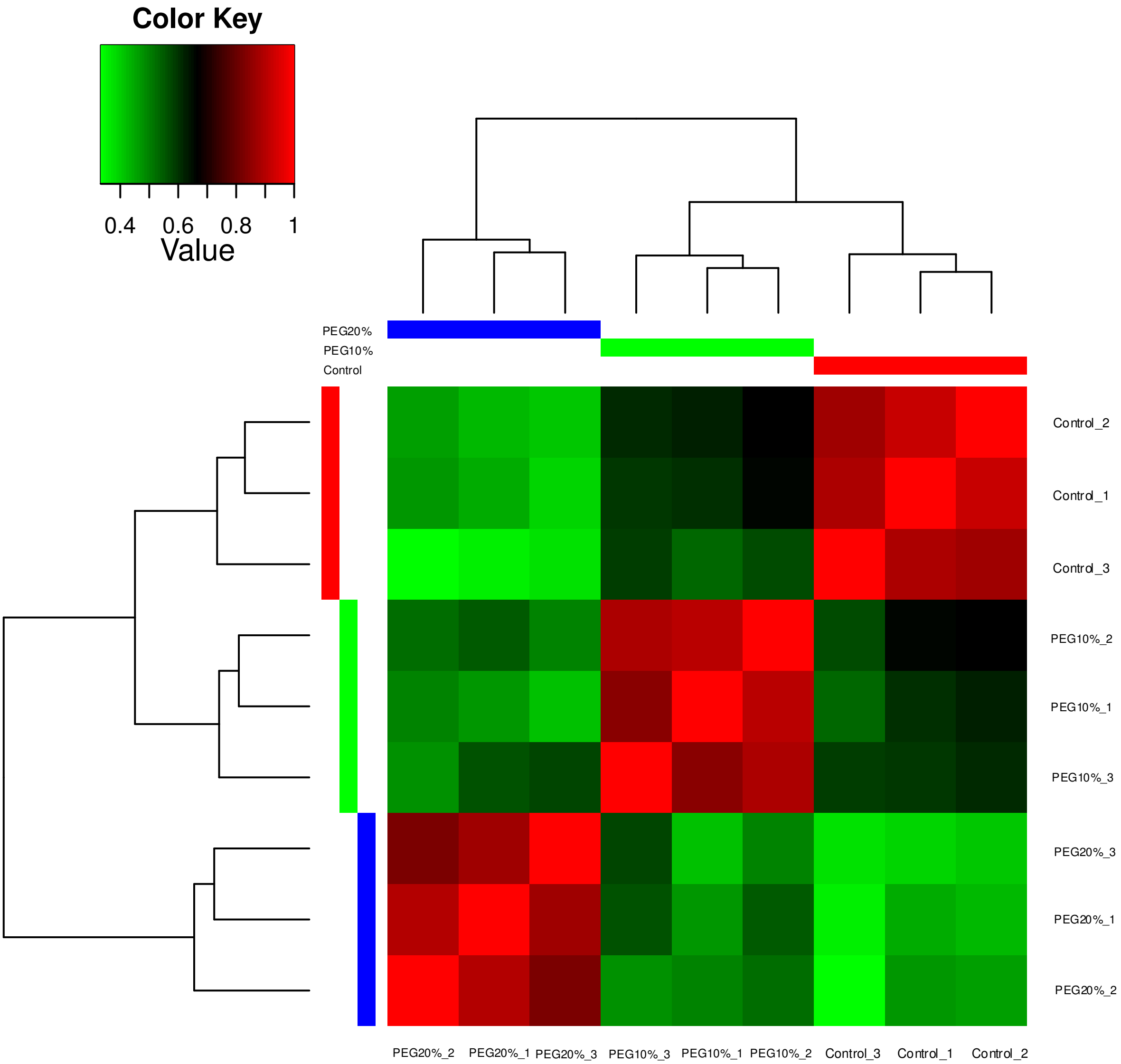
Color Key
0.4
0.6
0.8
1
Value
PEG20%
PEG10%
Control
Control_2
Control_1
Control_3
PEG10%_2
PEG10%_1
PEG10%_3
PEG20%_3
PEG20%_1
PEG20%_2
PEG20%_2
PEG20%_1
PEG20%_3
PEG10%_3
PEG10%_1
PEG10%_2
Control_3
Control_1
Control_2

(d)
